# Associations of vitamin D pathway genes with circulating 25-hydroxyvitamin-D, 1,25-dihydroxyvitamin-D, and prostate cancer: a nested case–control study

**DOI:** 10.1007/s10552-014-0500-5

**Published:** 2014-12-09

**Authors:** Rebecca Gilbert, Carolina Bonilla, Chris Metcalfe, Sarah Lewis, David M. Evans, William D. Fraser, John P. Kemp, Jenny L. Donovan, Freddie C. Hamdy, David E. Neal, J. Athene Lane, George Davey Smith, Mark Lathrop, Richard M. Martin

**Affiliations:** 1 School of Social and Community Medicine, University of Bristol, Canynge Hall, 39 Whatley Road, Bristol, BS8 2PS UK; 2 MRC Integrative Epidemiology Unit, University of Bristol, Bristol, UK; 3 Norwich Medical School, University of East Anglia, Norwich, UK; 4 Nuffield Department of Surgery, University of Oxford, Oxford, UK; 5 Department of Oncology, University of Cambridge, Cambridge, UK; 6 Commissariat à l’Energie Atomique, Center National de Génotypage, Evry, France; 7 Génome Québec Innovation Centre, McGill University, Montreal, Canada; 8 Bristol Biomedical Research Unit in Nutrition, National Institute for Health Research, Bristol, UK

**Keywords:** Prostate cancer, Vitamin D, Vitamin D pathway genes, 25 hydroxyvitamin-D, 1,25-dihydroxyvitamin-D

## Abstract

**Purpose:**

Vitamin D pathway single nucleotide polymorphisms (SNPs) are potentially useful proxies for investigating whether circulating vitamin D metabolites [total 25-hydroxyvitamin-D, 25(OH)D; 1,25-dihydroxyvitamin, 1,25(OH)_2_D] are causally related to prostate cancer. We investigated associations of sixteen SNPs across seven genes with prostate-specific antigen-detected prostate cancer.

**Methods:**

In a nested case–control study (within the ProtecT trial), we estimated odds ratios and 95 % confidence intervals (CIs) quantifying associations between SNPs and prostate cancer. Subgroup analyses investigated whether associations were stronger in men who had high/low sun exposure [a proxy for 25(OH)D]. We quantified associations of SNPs with stage (T1–T2/T3–T4) and grade (<7/≥7). Multiple variant scores included SNPs encoding proteins involved in 25(OH)D synthesis and metabolism.

**Results:**

We included 1,275 prostate cancer cases (141 locally advanced, 385 high grades) and 2,062 healthy controls. Vitamin D-binding protein SNPs were associated with prostate cancer (rs4588-A: OR 1.20, CI 1.01, 1.41, *p* = 0.04; rs7041-T: OR 1.19, CI 1.02, 1.38, *p* = 0.03). Low 25(OH)D metabolism score was associated with high (vs low) grade (OR 0.76, CI 0.63, 0.93, *p* = 0.01); there was a similar association of its component variants: rs6013897-A in *CYP24A1* (OR 0.78, CI 0.60, 1.01, *p* = 0.06) and rs10877012-T in *CYP27B1* (OR 0.80, CI 0.63, 1.02, *p* = 0.07). There was no evidence that associations differed by level of sun exposure.

**Conclusion:**

We found some evidence that vitamin D pathway SNPs were associated with prostate cancer risk and grade, but not stage. There was no evidence of an association in men with deficient vitamin D (measured by having low sun exposure).

**Electronic supplementary material:**

The online version of this article (doi:10.1007/s10552-014-0500-5) contains supplementary material, which is available to authorized users.

## Introduction

Prostate cancer is the most common male cancer in industrialized countries but knowledge of modifiable risk factors is limited. Metabolites of vitamin D [total 25-hydroxyvitamin-D, 25(OH)D; 1,25-dihydroxyvitamin, 1,25(OH)_2_D] control cellular growth and differentiation [1, 2], and administration of vitamin D analogues inhibits the progression of prostate cancer in animal models [3, 4] and in phase II trials [5]. In line with ecological studies and our understanding of the biological actions of vitamin D, other epidemiological studies have shown inverse associations of circulating total 25(OH)D with prostate cancer risk [1, 6, 7]. Overall, however, the evidence is inconsistent, with our recent meta-analyses finding little evidence that either increased life course sun exposure, dietary vitamin D, or circulating 25(OH)D or 1,25(OH)_2_D are associated with prostate cancer risk [8–10]. In line with some other studies [10], recent results from the cohort reported in this paper found a two-fold increased risk of more aggressive (locally advanced stage and/or high grade) prostate cancers in men with deficient (vs adequate) circulating 25(OH)D [11], but no association of 1,25(OH)_2_D with prostate cancer risk, stage, or grade [12].

In Mendelian randomization analyses, genetic variants can be used as proxy measures of nutritional exposure to determine the unconfounded and unbiased effect of modifiable risk factors on disease outcomes since they are not subject to the biases commonly found in observational studies (e.g., reverse causation, recall bias, confounding) [13, 14]. SNPs that are located in genes related to vitamin D metabolism and signalling could lend support to the hypothesis that vitamin D is related to prostate cancer and thus improve the case for a causal relationship. Several genetic variants regulate or influence the levels or actions of our exposure of interest [circulating 25(OH)D and 1,25(OH)_2_D]. Thus, if the intermediate phenotype is causally associated with the outcome (prostate cancer) then we would expect the genetic variants to be associated with the outcome, to the extent that the genetic variants affect the intermediate phenotype. There are a number of single nucleotide polymorphisms (SNPs) involved in the vitamin D pathway which are potentially useful proxies for investigating whether circulating vitamin D is causally related to prostate cancer: vitamin D 25-hydroxylase enzyme (*CYP2R1*) converts provitamin D (from sun exposure or dietary intake) into circulating 25(OH)D [15]; 1-α-hydroxylase (*CYP27B1*) converts 25(OH)D into 1,25(OH)_2_D (the active form of the hormone) [16]; circulating 1,25(OH)_2_D is degraded by 24-hydroxylase (*CYP24A1*) [16] to 24,25(OH)_2_D; vitamin D-binding protein (*VDBP* or *GC*) is the major carrier of 25(OH)D and 1,25(OH)_2_D, transporting the metabolites to the target tissue [17]; and the vitamin D-receptor gene (*VDR*) is a key mediator of the biological actions of 1,25(OH)_2_D [2]. VDR SNPs have not been found to be associated with circulating vitamin D levels in previous studies, but we include these SNPs in our analysis as components of the vitamin D pathway that could have an influence on cancer, including prostate cancer, despite not being associated with circulating vitamin D levels [18]. Recent genome-wide association studies (GWAS) have uncovered robust associations of 25(OH)D concentration with polymorphisms in the genetic variants in *VDBP*, *CYP2R1*, *CYP24A1,* and a region on chromosome 11 encompassing the genes for the 7-dehydrocholesterol reductase (*DHCR7*) and NAD synthetase 1 (*NADSYN*1) [19, 20]. Within this region, *DHCR7* is the most obvious candidate encoding an enzyme that catalyzes the conversion of 7-dehydrocholesterol (a precursor of vitamin D) to cholesterol.

We investigated associations of vitamin D pathway polymorphisms with PSA-detected prostate cancer, overall and stratified by stage and grade, in a large UK-wide population-based case–control study [21]. Few studies involve exclusively PSA-detected prostate cancer, an important factor in the PSA-era due to the increasingly earlier detection of localized disease. We hypothesized that polymorphisms that reflect lower levels or cellular uptake of total 25(OH)D or 1,25(OH)_2_D are associated with an increased risk of prostate cancer and that the association is stronger for locally advanced versus localized, and high-grade versus low-grade cancer. Given previous reports [10, 11, 22–24], we also investigated the possibility of a gene–environment interaction, i.e., whether the association is stronger in men who have a low level of sun exposure (as a proxy for deficient vitamin D status). We hypothesized that, among men with deficient vitamin D levels, those who have a genotype which improves cellular vitamin D status would have a lower risk of prostate cancer than men without this genotype, whereas this association would not be as strong in men with sufficient levels.

## Materials and methods

### Participants

The study is nested within a multi-center randomized controlled trial of treatments for localized disease: the Prostate Testing for cancer and Treatment (ProtecT) study [11, 21]. During recruitment to the ProtecT study (between 2001 and 2009), over 100,000 men aged 50–69 years at 337 general practices in nine UK centers (Birmingham, Bristol, Cambridge, Cardiff, Edinburgh, Leeds, Leicester, Newcastle, Sheffield) were offered a PSA test at a community-based ‘prostate check clinic’, and those with raised levels (≥3 ng/mL) were offered diagnostic biopsy. Detected tumors were all histologically confirmed and clinically staged using the TNM system [25]. Cancer stages T1–T2 were categorized as ‘localized’ and T3–T4 as ‘locally advanced’ as there were very few T4/metastasized tumors. ‘High-grade’ tumors were defined as a Gleason score ≥7 and low-grade tumors as Gleason score <7, after review of biopsy cores by a specialist uro-pathologist.

### Case–control selection

A total of 1,914 cases and 48,692 controls were potentially eligible for selection for the current study (based on men recruited between 2003 and 2008), had provided a plasma sample, and consented to prostate cancer research. We randomly selected one stratum-matched control for each case from those men who had provided a non-fasted blood sample at the prostate check clinic. Controls were randomly selected from the same stratum—i.e., 5-year age band (age at PSA test) and GP/family practice—as cases. Prostate check clinics were held over consecutive weeks at each GP practice, and so matching cases and controls by GP also matches on time and season of blood draw. All participants in the ProtecT prostate check clinics who had no evidence of prostate cancer were eligible for selection as controls; that is, men with a PSA test <3 ng/mL, or a raised PSA (≥3 ng/mL) combined with at least one negative biopsy and no subsequent prostate cancer diagnosis during the follow-up protocol for negative biopsies. All men provided written informed consent prior to inclusion in the study. Trent Multicentre Research Ethics Committee (MREC) approved the ProtecT study (MREC/01/4/025) and the associated ProMPT study which collected biological material (MREC/01/4/061).

### Vitamin D pathway genes and vitamin D assays

The following genes were genotyped in ProtecT participants as part of a genetic association study examining the effect of 70 diet/nutrition relevant SNPs on prostate cancer risk [18, 26]: *VDR* (*ApaI*: rs7975232, *BsmI*: rs1544410, *FokI*: rs10735810, *TaqI*: rs731236, *Cdx2*: rs11568820); *VDBP* (rs4588, rs7041); and *CYP27B1* (rs10877012). DNA extraction was performed by Tepnel (http://www.tepnel.com), and genotyping was undertaken by KBioscience Ltd (www.kbioscience.co.uk), who use their own form of competitive allele-specific PCR (KASPar) and Taqman™, for SNP analysis. Samples with more than 10 % genotype failure (7 SNPs) were defined as having poor DNA quality (2.6 %) and dropped from further analysis. Genotyping was repeated in 10 % of the study samples (with independent assessment) and for 99.98 % of those samples there was exact agreement between the two.

The remaining vitamin D pathway SNPs (CYP2R1: rs10741657, rs2060793; CYP24A1: rs6013897; DHCR7: rs12785878; NADSYN1: rs3829251; VDBP: rs2282679, rs1155563; CYP27B1: rs703842) were obtained from genome-wide genotyping of ProtecT samples, carried out on 3,390 individuals [27] at the Center National de Génotypage (Evry, France), using the Illumina Human660W-Quad_v1_A array (Illumina Inc.). The quality control process done before imputation excluded individuals on the basis of the following: sex mismatches, minimal (<0.325) or excessive heterozygosity (>0.345), disproportionate levels of individual missingness (>3 %), cryptic relatedness measured as proportion of identity by descent (IBD > 0.1), and insufficient sample replication (IBD < 0.8). The remaining individuals were assessed for evidence of population stratification by multidimensional scaling analysis and compared with HapMap II (release 22) European descent (CEU), Han Chinese (CHB), Japanese (JPT), and Yoruba (YRI) reference populations; all individuals with non-European ancestry were removed. SNPs with a minor allele frequency below 1 %, a call rate of <95 % or evidence for violations of Hardy–Weinberg equilibrium (*p* < 5 × 10^−7^), were discarded.

Circulating concentrations of total 25(OH)D (ng/mL) and 1,25(OH)_2_D (pg/mL) were measured in blood plasma collected at the prostate check clinic, prior to diagnosis, as described previously [11, 28]. Briefly, 25(OH)D_2_ and 25(OH)D_3_ samples were measured using tandem mass spectrometry, in 31 batches over a period of approximately 3 months [11], and 1,25(OH)_2_D samples were quantified by immunoassay [28] over a 2 months period using a single batch of reagents. Vitamin D levels were measured blind to diagnosis. Circulating concentrations of 25(OH)D_2_ and 25(OH)D_3_ were measured in nanograms per milliliter (ng/mL) where 1 ng/mL = 2.5 nmol/L (nanomoles per liter), and 1,25(OH)_2_D was measured in picomoles per liter (pmol/L) where 1 pg/mL = 2.6 pmol/L. Total 25(OH)D (ng/mL) was calculated as the summation of 25(OH)D_2_ and 25(OH)D_3._

### Vitamin D pathway scores

Multiple variant allele scores were created based on SNPs found to be associated with vitamin D status in prior studies [29] and in the current study [30]. Two scores were calculated by summing up all appropriate SNPs in each individual: (1) Synthesis score: genes encoding proteins involved in 25(OH)D synthesis (*CYP2R1* rs10741657, *DHCR7* rs12785878) [29]; and (2) Metabolism score: genes encoding proteins involved in 25(OH)D metabolism (*CYP24A1* rs6013897, *CYP27B1* rs10877012) [29]. Each SNP genotype was coded as 0, 1, or 2 depending on the number of risk alleles the individual carries and their effects on vitamin D levels calculated so that an increasing score indicates decreasing levels of vitamin D. If there were missing SNP data, the individual was given a missing score.

### Covariates

Measures of height, weight, weekly exercise, smoking status, family history of prostate cancer, history of benign prostatic hyperplasia, diabetes, occupational social class, and self-reported ethnicity were collected at the time of the initial PSA test [31], prior to knowledge of the PSA level or diagnosis in 85 % of men. We calculated body mass index (BMI; kg/m^2^), which represents general adiposity. A measure of ‘‘intense sun exposure’’ was derived by summing time spent sunbathing, on holiday, and in foreign countries, from birth up until 2 years prior to the prostate clinic [8]. Missing answers were considered as zero; however, scores were not calculated if more than half of answers were missing. These sun exposure questions have been analyzed in detail previously, and further details of their derivation are published [8].

To avoid bias caused by complete case analysis [32], we multiply imputed all missing covariate values (*i* = 10) using chained equations [33], assuming those values could be predicted without bias from the observed relationships between covariates and the outcome measure, and substituting imputed values for missing values. The proportion of missing values per covariate was: age-group 0 %, ethnicity 0.3 %, BMI 28 %, smoking 26 %, family history of prostate cancer 11 %, history of BPH 2 %, diabetes 30 %, social class 6 %, and intense sun exposure 48 %.

### Statistical analysis

#### Vitamin D pathway SNPs and scores and circulating 25(OH)D and 1,25(OH)_2_D

Genotypes were checked for deviation from Hardy–Weinberg equilibrium using the hwsnp function implemented in Stata (Stata Corporation, College Station, Texas). Linear regression was used to examine the association of 25(OH)D and 1,25(OH)_2_D with individual SNPs and genetic scores, assuming an additive genetic model. Analyses were adjusted by age, study center, and season of blood draw.

#### Vitamin D pathway SNPs and scores and prostate cancer risk

To allow for the matched sets of cases and controls, conditional logistic regression was used to estimate odds ratios (OR) and 95 % confidence intervals (CIs) quantifying the association between exposure and all prostate cancers. The model included the case–control matching variables, age, and GP/family practice. A case-only analysis used unconditional logistic regression, controlling for age, study center, and season of blood draw (i.e., to reflect the matching variables), to quantify associations of SNPs with prostate cancer stage (locally advanced vs localized) and grade [high (≥7) vs low (<7)]. A case-only analysis was used as all cases have undergone biopsy, therefore removing potential detection bias which could otherwise occur through misclassification of control status because of imperfect sensitivity of the PSA test [34]. SNPs were included as single variants, and effects were estimated per change in allele.

#### Gene–environment interaction of SNPs and scores and levels on prostate cancer

**Fig. 1:**
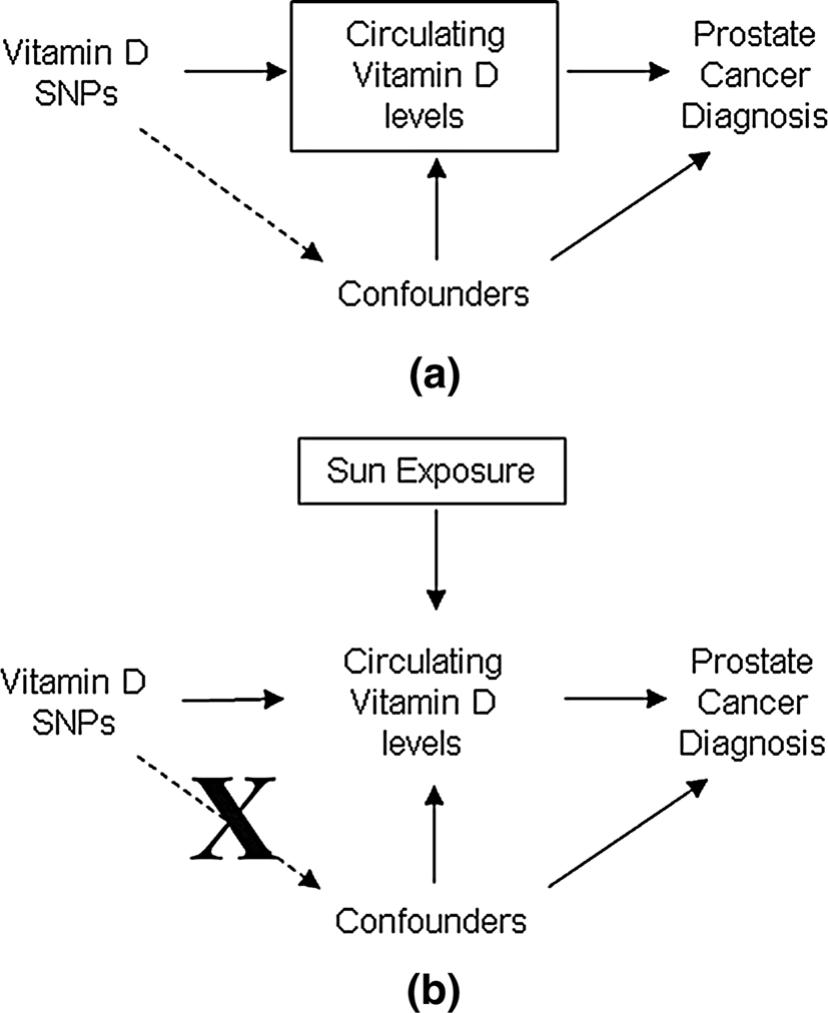
Diagram describing collider bias. **a** If we condition on circulating vitamin D levels (*box*), we could create on association of SNPs with confounders (*dashed line*). **b** If we condition on sun exposure (*box*), the association between SNPs and confounders is not generated because the SNPs are not associated with sun exposure (*dashed line* is removed)

The association of SNPs with prostate cancer was repeated, stratified by level of sun exposure. Since serum vitamin D level is an effect common to vitamin D pathway SNPs and confounders, attempting to estimate this association using serum vitamin D levels would have resulted in biased estimates which may have led to spurious associations between SNPs and prostate cancer risk (this is known as collider bias [35], Fig. 1). Instead, we used sun exposure as a proxy for high or low vitamin D level due to environmental factors, assuming that men with low sun exposure will tend to have lower vitamin D levels and that sun exposure was not associated with vitamin D pathway SNPs. The association of sun exposure with serum vitamin D level and SNPs were tested using *t* tests and Chi-squared tests, respectively.

A likelihood ratio test, comparing the main effects model with the model including an interaction term between the SNP and level of sun exposure, was used to calculate a *p* value for interaction. A dichotomized indicator of sun exposure was created based on levels above and below the median level of sun exposure.

### Population stratification

The top 10 principal components (PCs) that reflect the population’s genetic structure were estimated according to Price et al. [36] from genome-wide SNPs genotyped, imputed and cleaned as described above. All 10 PCs were included as covariates in all regression models to account for confounding by population stratification.

All analyses were carried out in Stata 12 (StataCorp, 2012. College Station, TX). We used ice for multiple imputation with chained equations [33] for imputing missing data. All tests of statistical significance were two-sided.

## Results

### Characteristics of study participants

**Fig. 2:**
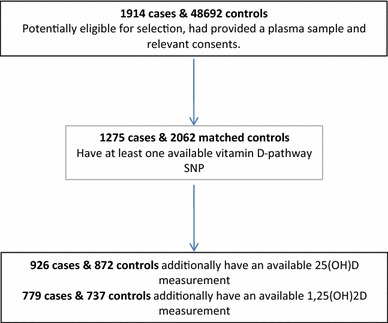
Flowchart describing the case–control selection for inclusion in this analysis

The current analysis includes 1,275 prostate cancer cases [1,131 (88.7 %) localized, 141 (11.1 %) locally advanced, 3 (0.24 %) missing stage; 887 (69.6 %) low grade, 385 (30.2 %) high grade, 3 (0.24 %) missing grade], and 2,062 controls that have at least one available SNP. 39.3 % of participants had data available on all SNPs, and 43 % were missing data on three or fewer SNPs. No man was missing more than 10 SNPs. Of these, 926 cases and 872 controls had an available 25(OH)D measurement and 779 cases and 737 controls had an available 1,25(OH)_2_D measurement (Fig. 2). The mean age of cases was 62.6 years and of controls was 61.7 years. As expected, the mean PSA level in cases was higher than in controls (9.5 vs 1.0 ng/mL). There were no substantial differences in baseline characteristics between cases and controls, except that more cases had a family history of prostate cancer versus controls (8.2 vs 5.6 %) and more cases had a normal (18.5–25) BMI (30.1 vs 25.5 %). Of the 99.3 % of subjects, who had recorded ethnicity, 98.9 % self-identified as white.

The mean (SD) 25(OH)D concentration in cases was 23.7 ng mL (8.7) and in controls was 23.5 ng/mL (8.7) (*p* for difference = 0.62). The mean (SD) 1,25(OH)_2_D concentration in cases was 40.6 pg.mL (18.4) and in controls was 40.9 pg/mL (18.1) (*p* for difference = 0.74). There were no differences between mean storage times of blood samples between cases and controls. Only *VDR* SNP rs7975232 was out of Hardy–Weinberg equilibrium in controls (*p* = 0.01).

### Vitamin D pathway SNPs and covariates

For the most part, there was no evidence of associations between vitamin D pathway SNPS and the covariates (data not shown). There was evidence of an association of *VDBP* rs4588-A with BMI (*p* = 0.01), *CYP27B1* rs703842-G with smoking status (*p* = 0.02), diabetes (*p* = 0.01) and social class (*p* = 0.01) and *CY27B1* rs10877012-T with smoking status (*p* = 0.01), and diabetes (*p* = 0.01). These associations are possibly due to chance as we carried out multiple tests, i.e., we tested 16 SNPs against eight covariates.

### Vitamin D pathway SNPs and circulating 25OHD and 1,25(OH)_2_D

There were up to 1,778 individuals with genome-wide SNP data and 25(OH)D and 1,25(OH)_2_D measurements. Of the 16 SNPs investigated, two SNPs in *CYP2R1* (rs10741657-A, rs2060793-A) and two SNPs in *VDR* (Fokl-A, Taql-C) were associated with 25(OH)D concentrations (with ss ranging from ≤0.001 to 0.04) (Table 1). One SNP in *DHCR*7 (rs12785878-G) and four SNPs in *VDR* (Apal-C, Bsml-A, Taql-C, Cdxl-A) were associated with 1,25(OH)_2_D concentrations (with *p* values ranging from 0.01 to 0.04) (Table 1). Four SNPs in *VDBP* (rs2282679-G, rs4588-A, rs7041-T, rs1155563-C) were associated with both 25(OH)D and 1,25(OH)_2_D concentrations (all *p* values ≤0.01) (Table 1). The above analyses were limited to controls only and were adjusted for age, study center, season of blood draw, and PCs. Nevertheless, associations of SNPs with 25(OH)D and 1,25(OH)_2_D concentrations did not differ by case–control status (results not shown). Based on the proportion of trait variability explained and on the *F*-statistic, which is related to the strength of the instrument, the best instruments for 25(OH)D concentrations in this population were *VDBP* SNPs (*F* = 12.2–22.3). Strong associations were detected between all *VDBP* SNPs and 1,25(OH)_2_D (*F* = 5.88–9.62). However, each polymorphism explained only ~1 % of the trait variance, and the *F*-statistics were below 10, the conventional lower limit of a strong instrument [30].

**Table 1:** The effects of vitamin D pathway SNPs on 25(OH)D (ng/mL) and 1,25(OH)_2_D (pg/mL) concentrations in control subjects

Polymorphism	Chromosome	Major/minor allele	MAF	Effect of minor allele on 25(OH)D (ng/mL)^a^	95 % CI	*p* value	*n*	*F*-statistic^b^	*R* ^2^ (%)	Effect of minor allele on 1,25(OH)_2_D (pg/ml)^a^	95 % CI	*p* value	*n*	*F*-statistic^b^	*R* ^2^ (%)
CYP2R1 rs10741657	11p15.2	G/A	0.43	0.92	(0.02, 1.83)	0.04	697	4.71	0.70	0.16	(−1.97, 2.28)	0.89	592	0.03	0.00
CYP2R1 rs2060793	11p15.2	G/A	0.43	0.92	(0.02, 1.83)	0.04	697	4.71	0.70	0.16	(−1.97, 2.28)	0.89	592	0.03	0.00
DHCR7 rs12785878	11q13.4	T/G	0.22	0.11	(−1.05, 1.26)	0.86	697	0.02	0.00	−3.85	(−6.58, −1.12)	0.01	592	5.81	1.00
NADSYN1 rs3829251	11q13.4	G/T	0.13	0.29	(−1.09, 1.68)	0.68	697	0.03	0.00	−2.31	(−5.59, 0.96)	0.17	592	1.31	0.20
*Synthesis score* ^c^		High–low		−0.56	(−1.28, 0.17)	0.13	697	3.31	0.50	−1.63	(−3.35, 0.10)	0.06	592	2.69	0.50
VDBP rs2282679	4q12	T/G	0.30	−2.27	(−3.23, −1.30)	<0.001	697	17.45	2.40	−3.29	(−5.61, −0.98)	0.01	592	8.01	1.30
VDBP rs4588	4q12	C/A	0.29	−2.14	(−3.12, −1.17)	<0.001	687	22.26	2.50	−3.17	(−5.49, −0.85)	0.01	584	8.17	1.10
VDBP rs7041	4q12	G/T	0.44	−1.65	(−2.53, −0.77)	<0.001	689	16.31	1.90	−2.72	(−4.82, −0.62)	0.01	586	5.88	0.80
VDBP rs1155563	4q12	T/C	0.30	−1.88	(−2.85, −0.91)	<0.001	697	12.21	1.70	−3.41	(−5.71, −1.11)	0.004	592	9.62	1.60
CYP24A1 rs6013897	20q13	T/A	0.20	−0.25	(−1.37, 0.87)	0.67	697	0.18	0.00	−1.62	(−4.26, 1.03)	0.23	592	0.84	0.10
CYP27B1 rs703842	12q13.1–13.3	A/G	0.33	−0.06	(−1.01, 0.89)	0.91	697	0.01	0.00	−0.17	(−2.38, 2.05)	0.88	592	0.00	0.00
CYP27B1 rs10877012	12q13.1–13.3	G/T	0.33	0.06	(−0.91, 1.03)	0.90	675	0.00	0.00	−0.20	(−2.45, 2.04)	0.86	574	0.16	0.00
*Metabolism score* ^d^		High–low		−0.02	(−0.78, 0.73)	0.95	672	0.02	0.00	−0.72	(−2.49, 1.05)	0.42	571	0.27	0.00
VDR ApaI (rs7975232)	12q13.11	A/C	0.48	−0.46	(−1.32, 0.40)	0.29	681	0.88	0.10	−2.39	(−4.41, −0.38)	0.02	580	4.79	0.70
VDR BsmI (rs1544410)	12q13.11	G/A	0.40	0.85	(−0.06, 1.77)	0.07	684	3.16	0.40	2.62	(0.50,4.75)	0.02	580	3.85	0.50
VDR FokI (rs10735810)	12q13.11	G/A	0.38	−1.26	(−2.19, −0.32)	0.01	688	6.30	0.70	0.88	(−1.30, 3.06)	0.43	584	0.57	0.10
VDR TaqI (rs731236)	12q13.11	T/C	0.40	0.98	(0.06, 1.90)	0.04	691	3.85	0.40	2.26	(0.13, 4.40)	0.04	589	3.42	0.50
VDR Cdx2 (rs11568820)	12q13.11	G/A	0.21	−0.33	(−1.43, 0.76)	0.55	689	0.86	0.10	3.73	(1.12, 6.33)	0.01	586	4.59	0.60

*MAF* minor allele frequency, *CI* confidence interval

^a^Difference in mean levels, calculated using regression, adjusting for exact age, study center, season of blood draw and 10 principal components to account for confounding by population stratification

^b^ *F*-statistic and *R*-squared (*R* ^2^) indicate how much of the variability in vitamin D levels is explained by each SNP (i.e., the strength of each SNP as an instrument for vitamin D levels). *F* > 10 indicates a strong instrument. Calculated using regression, unadjusted

^c^Synthesis score: rs10741657, rs12785878

^d^Metabolism score: rs6013897, rs10877012

### Vitamin D pathway SNPs and prostate cancer risk

There was evidence of an association of linked *VDBP* SNPs rs4588-A and rs7041-T, representing low levels of 25(OH)D, with prostate cancer risk (rs4588: OR 1.20, 95 % CI 1.01, 1.41, *p* = 0.04; rs7041: OR 1.19, 95 % CI 1.02, 1.38, *p* = 0.03). There was no evidence that the other SNPs or scores were associated with prostate cancer risk (Table 2).

**Table 2:** Associations between vitamin D pathway SNPs and prostate cancer risk (cases vs controls)

Polymorphism	Major/minor allele	25(OH)D-increasing allele^a^	*n* (cases/controls)			Effect of minor allele^b^		
			Major homozygote	Heterozygote	Minor homozygote	OR	(95 % CI)	*p* value
CYP2R1 rs10741657	G/A	A	349/637	485/887	204/351	1.00	(0.90, 1.13)	0.94
CYP2R1 rs2060793	G/A	A	349/637	485/887	204/351	1.00	(0.90, 1.13)	0.94
DHCR7 rs12785878	T/G	T	621/1177	372/601	45/97	1.07	(0.93, 1.23)	0.32
NADSYN1 rs3829251	G/T	A	790/1418	233/421	15/36	0.97	(0.82, 1.15)	0.75
*Synthesis score* ^c^	High–low					1.03	(0.94, 1.12)	0.56
VDBP rs2282679	T/G	T	504/948	433/757	101/170	1.09	(0.96, 1.23)	0.17
VDBP rs4588	C/A	C	461/465	400/358	92/75	1.20	(1.01, 1.41)	0.04
VDBP rs7041	G/T	G	275/295	473/430	201/175	1.19	(1.02, 1.38)	0.03
VDBP rs1155563	T/C	T	513/955	421/741	104/179	1.04	(0.92, 1.18)	0.52
CYP24A1 rs6013897	T/A	T	665/1220	348/577	25/78	1.01	(0.87, 1.17)	0.92
CYP27B1 rs703842	A/G	A	456/866	466/814	116/195	1.06	(0.93, 1.19)	0.38
CYP27B1 rs10877012	G/T	G	405/413	408/377	111/91	1.02	(0.86, 1.20)	0.85
*Metabolism score* ^d^	High–low					0.97	(0.85, 1.10)	0.6
VDR ApaI	A/C	A	259/251	489/419	202/220	0.94	(0.81, 1.10)	0.45
VDR BsmI	G/A	A	342/334	468/423	144/138	1.07	(0.91, 1.25)	0.41
VDR FokI	G/A	G	353/339	486/425	111/131	1.06	(0.90, 1.25)	0.51
VDR TaqI	T/C	C	345/339	465/433	144/130	1.06	(0.91, 1.25)	0.45
VDR Cdx2	G/A	G	599/560	296/298	56/40	1.03	(0.86, 1.24)	0.72

*OR* odds ratio, *CI* confidence interval

^a^Determined from ProtecT data (presented in Table 1)

^b^Case versus control models are calculated using conditional logistic regression, stratum matched on 5 years age band and GP surgery. Models additionally included exact age and 10 principal components to account for confounding by population stratification

^c^Synthesis score: rs10741657, rs12785878

^d^Metabolism score: rs6013897, rs10877012

There was no convincing evidence that either the vitamin D pathway SNPs or the two scores were associated with stage (Table 3).There was evidence that the metabolism score, indicating decreasing 25(OH)D levels, was associated with Gleason grade (high vs low) (OR 0.76, 95 % CI 0.63, 0.93, *p* = 0.01), and marginal evidence for a similar association of its component variants rs6013897-A in *CYP24A1* (OR 0.78, 95 % CI 0.60, 1.01, *p* = 0.06) and rs10877012-T in *CYP27B1* (OR 0.80, 95 % CI 0.63, 1.02, *p* = 0.07) (representing low levels of 25(OH)D).

**Table 3:** Associations between vitamin D pathway SNPs and prostate cancer stage and Gleason grade (case-only analyses)

Polymorphism	Major/minor allele	25(OH)D-increasing allele^b^	Stage: advanced versus localized^a^						Gleason grade: high versus low grade^a^					
			Major homo-zygote	Hetero-zygote	Minor homo-zygote	Effect of minor allele: OR	(95 % CI)	*p* value	Major homo-zygote	Hetero-zygote	Minor homo-zygote	Effect of minor allele: OR	(95 % CI)	*p* value
CYP2R1 rs10741657	G/A	A	41/308	48/436	24/180	1.03	(0.78, 1.37)	0.81	101/247	142/342	64/140	1.08	(0.89, 1.30)	0.45
CYP2R1 rs2060793	G/A	A	41/308	48/436	24/180	1.03	(0.78, 1.37)	0.81	101/247	142/342	64/140	1.08	(0.89, 1.30)	0.45
DHCR7 rs12785878	T/G	T	70/550	39/333	4/41	0.95	(0.66, 1.35)	0.76	187/433	106/265	14/31	0.94	(0.74, 1.19)	0.62
NADSYN1 rs3829251	G/T	A	88/701	23/210	2/13	0.99	(0.64, 1.53)	0.95	228/560	72/161	7/8	1.17	(0.88, 1.55)	0.29
*Synthesis score* ^c^	High–low					0.96	(0.77, 1.19)	0.71				0.94	(0.81, 1.08)	0.37
VDBP rs2282679	T/G	T	53/451	49/383	11/90	1.01	(0.74, 1.37)	0.94	143/359	126/307	38/63	1.15	(0.94, 1.41)	0.18
VDBP rs4588	C/A	C	45/416	51/346	11/81	1.17	(0.81, 1.69)	0.41	152/307	128/271	37/55	1.21	(0.95, 1.54)	0.12
VDBP rs7041	G/T	G	23/252	57/413	23/178	1.11	(0.78, 1.59)	0.56	81/194	156/314	74/127	1.19	(0.95, 1.49)	0.13
VDBP rs1155563	T/C	T	55/458	46/374	12/92	0.99	(0.73, 1.34)	0.95	142/369	128/293	37/67	1.17	(0.95, 1.43)	0.13
CYP24A1 rs6013897	T/A	T	75/590	32/315	6/19	1.04	(0.72, 1.51)	0.82	212/451	87/261	8/17	0.78	(0.60, 1.01)	0.06
CYP27B1 rs703842	A/G	A	53/403	46/419	14/102	0.91	(0.67, 1.24)	0.56	144/310	125/341	38/78	0.94	(0.76, 1.15)	0.53
CYP27B1 rs10877012	G/T	G	46/359	46/360	11/99	0.92	(0.63, 1.33)	0.65	151/252	116/292	37/73	0.8	(0.63, 1.02)	0.07
*Metabolism score* ^d^	High–low					0.82	(0.61, 1.10)	0.18				0.76	(0.63, 0.93)	0.01
VDR ApaI	A/C	A	31/227	53/434	23/179	1.12	(0.80, 1.59)	0.51	80/178	160/327	75/127	1.2	(0.96, 1.51)	0.11
VDR BsmI	G/A	A	37/304	53/414	17/126	1.04	(0.74, 1.47)	0.82	127/215	142/324	47/96	0.89	(0.71, 1.12)	0.32
VDR FokI	G/A	G	37/314	54/431	15/96	1.12	(0.78, 1.61)	0.54	104/247	174/311	35/76	1.15	(0.90, 1.46)	0.27
VDR TaqI	T/C	C	35/309	55/409	17/126	1.04	(0.74, 1.48)	0.8	127/218	144/319	46/97	0.89	(0.71, 1.12)	0.31
VDR Cdx2	G/A	G	57/539	38/258	11/45	1.30	(0.89, 1.89)	0.18	193/403	94/202	27/29	1.14	(0.88, 1.47)	0.33

Locally advanced stage is T3–T4, localized stage is T1–T2, N0, M0. High grade is Gleason grade ≥7, low grade is <7

*OR* odds ratio, *CI* confidence interval

^a^Case-only models are calculated using logistic regression, adjusted for exact age, season of blood draw, and study center location. Models additionally included 10 principal components to account for confounding by population stratification

^b^Determined from ProtecT data (presented in Table 1)

^c^Synthesis score: rs10741657, rs12785878

^d^Metabolism score: rs6013897, rs10877012

### Vitamin D pathway SNPs, sun exposure and prostate cancer risk

The mean 25(OH)D level in men who had below the median sun exposure was 22.1 ng/nL and who had above the median sun exposure was 24.6 ng/mL (*p* for difference <0.001). None of the vitamin D pathway SNPs were associated with sun exposure (data available on request). There was no evidence of an association between any SNPs or scores and prostate cancer risk within men with below the median sun exposure (all *p* interaction >0.04) (Table 4).

**Table 4:** Associations between SNPs and prostate cancer risk, in men with high and low sun exposure

Polymorphism	Major/minor allele	*n* (cases/controls)			Effect of minor allele^a^		*n* (cases/controls)			Effect of minor allele^a^		*p* interaction^b^
		Major homo-zygote	Hetero -zygote	Minor homo-zygote	OR	(95 % CI)	Major homo-zygote	Hetero -zygote	Minor homo- zygote	OR	(95 % CI)	
		Men with below the median sun exposure					Men with above the median sun exposure					
CYP2R1 rs10741657	G/A	163/239	221/363	92/130	1.00	(0.84, 1.18)	128/205	182/263	81/116	1.05	(0.87, 1.27)	0.55
CYP2R1 rs2060793	G/A	163/239	221/363	92/130	1.00	(0.84, 1.18)	128/205	182/263	81/116	1.05	(0.87, 1.27)	0.55
DHCR7 rs12785878	T/G	279/461	173/226	24/45	1.07	(0.87, 1.31)	233/363	143/196	15/25	0.99	(0.78, 1.26)	0.47
NADSYN1 rs3829251	G/T	352/558	118/161	6/13	1.06	(0.82, 1.36)	303/445	81/128	7/11	0.87	(0.65, 1.16)	0.28
*Synthesis score* ^c^	High–low				1.03	(0.90, 1.18)				0.97	(0.83, 1.12)	0.14
VDBP rs2282679	T/G	250/367	194/299	32/66	0.90	(0.75, 1.10)	183/319	163/216	45/49	1.14	(0.93, 1.40)	0.04
VDBP rs4588	C/A	210/208	189/168	32/41	1.06	(0.81, 1.40)	142/172	124/123	40/23	1.34	(1.10, 1.65)	0.08
VDBP rs7041	G/T	126/134	216/190	86/95	1.07	(0.84, 1.37)	84/110	155/157	70/52	1.55	(1.15, 2.08)	0.06
VDBP rs1155563	T/C	250/370	187/297	39/65	0.92	(0.76, 1.11)	192/315	159/213	40/56	1.66	(1.24, 2.22)	0.26
CYP24A1 rs6013897	T/A	311/483	153/221	12/28	0.93	(0.75, 1.16)	242/379	138/188	11/17	1.15	(0.90, 1.49)	0.52
CYP27B1 rs703842	A/G	207/345	210/310	59/77	1.12	(0.93, 1.35)	173/258	176/267	42/59	0.98	(0.80, 1.21)	0.35
CYP27B1 rs10877012	G/T	174/202	181/163	59/46	1.19	(0.93, 1.54)	141/131	126/144	26/30	0.87	(0.64, 1.19)	0.27
*Metabolism score* ^d^	High–low				1.03	(0.85, 1.26)				0.92	(0.73, 1.17)	0.61
VDR ApaI	A/C	128/112	216/192	84/108	0.90	(0.71, 1.14)	70/84	169/153	66/78	1.01	(0.76, 1.35)	0.47
VDR BsmI	G/A	150/163	219/188	63/63	1.11	(0.87, 1.42)	112/127	151/147	41/45	1.08	(0.79, 1.47)	0.86
VDR FokI	G/A	172/152	209/188	47/72	0.91	(0.70, 1.18)	103/127	159/153	46/40	1.34	(1.00, 1.78)	0.24
VDR TaqI	T/C	150/167	214/192	64/59	1.10	(0.86, 1.41)	115/128	154/151	40/43	1.04	(0.77, 1.42)	0.83
VDR Cdx2	G/A	271/265	135/134	24/18	1.08	(0.80, 1.45)	189/187	95/108	17/18	0.78	(0.56, 1.07)	0.46

*OR* odds ratio, *CI* confidence interval

^a^Case versus control models are calculated using conditional logistic regression, stratum matched on 5 years age band and GP surgery. Models additionally included exact age and 10 principal components to account for confounding by population stratification

^b^ *p* for interaction calculated using a likelihood ratio test comparing the main effects model with the model including an interaction term between the SNP and an indicator of vitamin D deficiency

^c^Synthesis score: rs10741657, rs12785878

^d^Metabolism score: rs6013897, rs10877012

### Population stratification

All analyses were adjusted for population stratification. There was no evidence of an association between each SNP and score with the principal components used in this adjustment, indicating that population stratification was not likely to have affected our results (Supplementary Table 1).

## Discussion

This study, of 1,275 prostate cancer cases and 2,062 healthy controls from the ProtecT study, investigated associations of sixteen vitamin D pathway polymorphisms with PSA-detected prostate cancer risk and, in cases, with stage and Gleason grade. There was evidence that two SNPs in *VDBP*, representing low 25(OH)D levels, were associated with increased prostate cancer risk and that a score measuring metabolism (indicating low 25(OH)D levels) and its component variants were associated with high Gleason grade. There was no other convincing evidence that vitamin D pathway SNPs were associated with prostate cancer risk, stage, or grade. There was no evidence that associations differed by level of sun exposure.

We validated the use of GWAS-identified SNPs in ProtecT as proxies for serum 25(OH)D as well as of scores including these variants, confirming associations reported in previous studies. The synthesis score appeared to be a reasonably strong instrument, although the scores explained less than 1 % of the trait variance for circulating 25(OH)D and 1,25(OH)_2_D. Four SNPs in *VDBP* (rs2282679, rs4588, rs7041, rs1155563) were strong instruments for 25(OH)D, and one SNP in *VDBP* (rs1155563) was a strong instrument for 1,25(OH)_2_D) (those SNPs with *F* ≥ 10), explaining approximately 2 % of the variability.

Results from the Health Professionals Follow-up Study (HPFS) found that variants in *CYP27A1* (*p* = 0.02) and *VDR* (12 SNPs, *p* = 0.01), and a score made up of seven vitamin D pathway genes (*CYP27A1, CYP2R1, CYP27B1, VDBP, CYP24A1, RXRA, VDR*. *p* = 0.008), were associated with risk of lethal prostate cancer [37]. There were no associations between prostate cancer risk and 212 SNPs from 12 genes related to vitamin D (including *CYP27A1, VDBP, CYP27B1, CYP24A1, VDR*) examined in the Prostate, Lung, Colorectal and Ovarian Cancer Screening Trial (749 incident cases, 781 controls) [23]. Among men in the lowest tertile of 25(OH)D, there was an association between three *VDR* SNPs (rs11574143, rs757343, *BsmI*) and prostate cancer risk (the strongest association was for rs11574143: OR 2.49, 95 % CI 1.51, 4.11). Results from the National Cancer Institute Breast and Prostate Cancer Cohort Consortium (BPC3), a pooled analysis of 10,000 cases and 11,000 controls, found that genetic variants near CYP24A1 were associated with a decreased risk of aggressive prostate cancer (*p* trend <0.001), and a score made of four genes thought to predict circulating levels of 25(OH)D (*VDBP*, *CYP24A1*, *CYP2R1*, *DHCR7*) was related to both overall and aggressive prostate cancer [38]. There was no association between prostate cancer risk and the other SNPs. Variants in the *VDR* have been associated with advanced stage or high Gleason grade [18, 39], with a recent meta-analysis of 13 studies, including data from this study, finding an association between three *VDR* polymorphisms (*ApaI*, *BsmI* and *TaqI*) and prostate cancer grade [18]. However, VDR SNPs have not been consistently associated with 25(OH)D or 1,25(OH)_2_D concentrations or with prostate cancer risk. A recent meta-analysis of 34 studies found no evidence of an association of *VDR BsmI* and *FokI* with prostate cancer risk [40]. *VDR Cdx2* AA genotype was associated with prostate cancer in men with low 25(OH)D (≤15 ng/mL) (*p* interaction = 0.02) and with aggressive and high-grade prostate cancer in men with low 25(OH)D and 1,25(OH)_2_D (*p* interaction = 0.04 and 0.01, respectively) compared with men with normal levels [24]. Results from the Physician’s Health Study [22] found no associations of *VDR* *BsmI* or *TaqI* polymorphisms and prostate cancer risk (372 incident cases, 591 controls), although in men with 25(OH)D below the median there was a 57–62 % reduction in risk of men with the *BsmI* *AA* genotype (RR 0.43, 95 % CI 0.19, 0.98) or the *TaqI* *CC* genotype (data not shown) compared with men with the *GG* or *TT* genotypes. The risk was reduced by 80–90 % in men aged over 61 years (*BsmI*: RR 0.18, 95 % CI 0.05, 0.68). Variants in *VDR, CYP24A1, and CYP27B1* were associated with progression to prostate cancer-specific mortality in a case-only study (*n* = 1,294) [41]. Our recent study found no evidence that circulating levels or vitamin D pathway genes (*VDR: ApaI, BsmI, FokI, TaqI, Cdx2*; *VDBP*: rs4588, rs7041; *CYP27B1*: rs10877012) influence PSA-defined progression in men with localized prostate cancer on active monitoring [42].

Our recent epidemiological study from the same cohort found a two-fold increased risk of more aggressive (locally advanced stage and/or high grade) prostate cancers in men deficient in circulating 25(OH)D (<12 ng/mL) [11]. However, none of the SNPs expected to modulate 25(OH)D were associated with prostate cancer risk, stage, or grade. SNPs in the *VDR*, a key mediator of the biological actions of 1,25(OH)_2_D [2], were not associated with stage or grade. Our recent study found no association between 1,25(OH)_2_D and prostate cancer risk,stage, or grade [12]. Circulating levels of 1,25(OH)_2_D are tightly regulated [1]. Biological evidence shows that the prostate can locally convert 25(OH)D to 1,25(OH)_2_D [43, 44], although prostate cancer tissue has a reduced ability to locally convert 25(OH)D to 1,25(OH)_2_D [45]. *VDR* is present in the prostate gland [43, 46], so *VDR* status may better indicate local 1,25(OH)_2_D status than circulating levels. Circulating levels may not be a good indicator of what is happening at the cellular level. Recent results from the Alpha-Tocopherol, Beta-carotene Cancer Prevention (ATBC), study found that their observed association between circulating vitamin D and prostate cancer risk was made stronger when vitamin D-binding protein concentrations were also elevated [47]. This suggests that VDBP may modulate the impact of vitamin D status on prostate cancer, even though the SNPs were not directly associated with prostate cancer risk overall. Two *VDBP* SNPs were associated with prostate cancer risk in the current study, although there was no evidence of an interaction when stratified by sun exposure (*p* interaction = 0.12).

### Strength and limitations of our study

Our study includes a large sample, with more men with prostate cancer and matched controls than previous studies, about which we have extensive information recorded. All of our men were resident in the UK and 99 % of our subjects self-reported their ethnicity as white. Since the decision to biopsy was based on PSA level, some of the controls with PSA <3 ng/mL will have unidentified prostate cancer [34] (misclassification bias) but this would not affect our analysis of locally advanced versus localized cancers (as all cancers were biopsy confirmed). Any misclassification of cancer status is likely to be non-differential with respect to vitamin D pathway polymorphisms, at most moderately attenuating any effect-estimates [48]. Our case-only comparison uses 25OHD and 1,25(OH)_2_D concentrations measured in men diagnosed with locally advanced/high-grade cancer versus men diagnosed whilst their tumor was localized/low grade. We categorized cancer stages T3–T4 as ‘locally advanced’ as there were very few T4/metastasized tumors [~6 % have distal metastasis (T4 or M1)].

Circulating 25(OH)D and 1,25(OH)_2_D concentrations were measured at one laboratory, in as few batches and in as short a time frame as possible (thus attenuating any potential technical errors of measurement). Circulating vitamin D levels were measured in plasma collected at the prostate check clinic prior to diagnosis, with measurement of vitamin D concentrations blind to diagnosis. The study is population-based and thus subject to little selection bias. Circulating levels of 25(OH)D may vary by season of tissue collection [49], which is not a problem when analyzing vitamin D pathway SNPs. It is possible that we are studying a relatively healthy population, within which there is not enough variation in vitamin D status to be able to detect an affect [25(OH)D: IQR in cases: 17.4,28.9 ng/mL, controls: 17.8,28.4 ng/mL; 1,25(OH)_2_D: IQR in cases = 27.5,51.1 pg/mL, controls: 27.8,50.8]. Circulating 25(OH)D and 1,25(OH)_2_D may not reflect the amount of 25(OH)D and 1,25(OH)_2_D available for use within the target tissues [50], so we may be using the wrong instruments to examine the effects of vitamin D status. Even though our sample was large, because the genetic variants explain a small proportion of the variability in circulating vitamin D levels, we would need an even larger sample to find robust evidence of an effect on prostate cancer and to be able to provide accurate estimates for this effect.

Our Mendelian randomization (MR) approach is more reliable than results from observational studies and can be used to strengthen the evidence of causality since genotypes are unlikely to be affected by confounding or reverse causation and are not subject to high levels of measurement error. Our SNPs and genetic scores were mostly not associated with the confounders, thus satisfying one of the main assumptions of MR. However, we did find evidence of an association of rs4588 with BMI and rs10877012 with smoking. These associations may be chance findings, but, if real, may pose a problem to our inference of causality, as BMI has been associated with prostate cancer [51]. All SNPs, except for the VDR SNPs, were assumed to be strong instruments based on previously published data, although in our control population only the VDBP SNPs were validated as such. Another important consideration in MR is that the instrument (i.e., the genetic score) should be associated with the outcome of interest (i.e., prostate cancer) only via the exposure (i.e., circulating vitamin D levels). For this assumption to hold the SNPs included in the genetic score cannot have pleiotropic effects on prostate cancer. This means that a genetic variant with biological pleiotropy will additionally affect prostate cancer via phenotypes unrelated to circulating vitamin D level. Since we cannot test the assumption of no effect of the instrument on the outcome via pathways other than through the exposure of interest, it is not possible to completely rule out pleiotropic influences on our results.

## Conclusion

Our study found evidence that two SNPs in vitamin D-binding protein were associated with prostate cancer risk (rs4588-A and rs7041-T). A score measuring metabolism, and its component variants (rs6013897-A in *CYP24A1* and rs10877012T in *CYP27B1*), were associated with Gleason grade (high grade vs low grade). There was no association of other vitamin D pathway polymorphisms being associated with overall prostate cancer risk, stage, or grade. There was no evidence of an association in men with deficient vitamin D (measured by having low sun exposure).

## Electronic supplementary material

Below is the link to the electronic supplementary material.

Supplementary material 1 (DOCX 44 kb)
