# Supplementary material for: Associations of vitamin D pathway genes with circulating 25-hydroxyvitamin-D, 1,25-dihydroxyvitamin-D, and prostate cancer: a nested case–control study
Source: Cancer Causes Control. 2014 Dec 9;26(2):205–18. doi: 10.1007/s10552-014-0500-5 (PMC4298668; doi:10.1007/s10552-014-0500-5)
Supplement: Supplementary file 1 — Supplementary material 1 (DOCX 44 kb) [file 10552_2014_500_MOESM1_ESM.docx]

Associations of vitamin D-pathway genes with circulating 25-hydroxyvitamin-D, 1,25-dihydroxyvitamin-D and prostate cancer:

Supplementary Table

Rebecca Gilbert^1^, Carolina Bonilla^1^, Chris Metcalfe^1^, Sarah Lewis^1,2^, David M Evans^2^, William D Fraser^3^, John P Kemp^1,2^, Jenny Donovan^1^, Freddie Hamdy^4^, David E Neal^5^, J Athene Lane^1^, George Davey Smith^1,2^*, Mark Lathrop^6,7^*, Richard M Martin^1,2,8^*

^1^ School of Social and Community Medicine, University of Bristol, Bristol, UK.

^2^ MRC Centre for Causal Analysis in Translational Epidemiology, School of Social and Community Medicine, University of Bristol, Bristol, UK.

^3^ Norwich Medical School, University of East Anglia, Norwich, UK.

^4^ Nuffield Department of Surgery, University of Oxford, Oxford, UK.

^5^ Department of Oncology, University of Cambridge, Cambridge, UK.

^6^ Commissariat à l'Energie Atomique, Center National de Génotypage, Evry, France.

^7^McGill University-Génome Québec Innovation Centre, Montreal, Canada

^8^National Institute for Health Research, Bristol Biomedical Research Unit in Nutrition, Bristol, UK

*Joint last authors – these authors contributed equally to this work.

**Corresponding author:** Rebecca Gilbert, School of Social and Community Medicine, University of Bristol, Canynge Hall, 39 Whatley Road, Bristol, BS8 2PS, UK. Email: Becky.Gilbert[@bristol.ac.uk](mailto:Chris.Metcalfe@bristol.ac.uk)

**Supplementary Table 1**: Associations between SNPs and the ten principal components (PCs) used to adjust all analyses for population stratification

| **PCs^a^** | **Effect of SNP on PC^b^** | **95% CI** | **p-value** | **Effect of SNP on PC^b^** | **95% CI** | **p-value** | **Effect of SNP on PC^b^** | **95% CI** | **p-value** |
| --- | --- | --- | --- | --- | --- | --- | --- | --- | --- |
|  | **CYP2R1 rs10741657** | | | **CYP2R1 rs2060793** | | | **DHCR7 rs12785878** | | |
| 1 | -0.00046 | (-0.00137,0.00044) | 0.314 | -0.00046 | (-0.00137,0.00044) | 0.314 | -0.00142 | (-0.00252,-0.00032) | 0.011 |
| 2 | 0.00036 | (-0.00055,0.00127) | 0.436 | 0.00036 | (-0.00055,0.00127) | 0.436 | -0.00035 | (-0.00145,0.00075) | 0.535 |
| 3 | -0.00079 | (-0.00170,0.00011) | 0.086 | -0.00079 | (-0.00170,0.00011) | 0.086 | -0.00027 | (-0.00137,0.00083) | 0.628 |
| 4 | -0.00021 | (-0.00112,0.00070) | 0.651 | -0.00021 | (-0.00112,0.00070) | 0.651 | -0.0006 | (-0.00171,0.00050) | 0.285 |
| 5 | 0.00053 | (-0.00037,0.00144) | 0.248 | 0.00053 | (-0.00037,0.00144) | 0.248 | -0.00033 | (-0.00144,0.00077) | 0.553 |
| 6 | -0.00078 | (-0.00169,0.00013) | 0.095 | -0.00078 | (-0.00169,0.00013) | 0.095 | 0.00102 | (-0.00009,0.00212) | 0.072 |
| 7 | 0.00043 | (-0.00047,0.00134) | 0.348 | 0.00043 | (-0.00047,0.00134) | 0.348 | -0.00034 | (-0.00144,0.00076) | 0.542 |
| 8 | -0.00029 | (-0.00120,0.00062) | 0.531 | -0.00029 | (-0.00120,0.00062) | 0.531 | -0.0015 | (-0.00260,-0.00039) | 0.008 |
| 9 | 0.00081 | (-0.00009,0.00172) | 0.079 | 0.00081 | (-0.00009,0.00172) | 0.079 | -0.00046 | (-0.00156,0.00065) | 0.419 |
| 10 | 0.00022 | (-0.00070,0.00113) | 0.642 | 0.00022 | (-0.00070,0.00113) | 0.642 | -0.00072 | (-0.00182,0.00039) | 0.206 |
|  |  |  |  |  |  |  |  |  |  |
|  | **NADSYN1 rs3829251** | | | **Synthesis score^c^** | | | **VDBP rs2282679** | | |
| 1 | -0.00164 | (-0.00299,-0.00029) | 0.017 | -0.0003 | (-0.00100,0.00040) | 0.405 | -0.00011 | (-0.00109,0.00088) | 0.831 |
| 2 | -0.00026 | (-0.00162,0.00109) | 0.704 | -0.00036 | (-0.00106,0.00034) | 0.318 | 0.0002 | (-0.00079,0.00119) | 0.698 |
| 3 | -0.00068 | (-0.00203,0.00067) | 0.326 | 0.00036 | (-0.00034,0.00107) | 0.308 | 0.00079 | (-0.00019,0.00178) | 0.115 |
| 4 | -0.0005 | (-0.00186,0.00086) | 0.469 | -0.00012 | (-0.00082,0.00059) | 0.740 | 0.00009 | (-0.00090,0.00108) | 0.856 |
| 5 | -0.00087 | (-0.00223,0.00048) | 0.206 | -0.00046 | (-0.00116,0.00025) | 0.203 | 0.00043 | (-0.00056,0.00141) | 0.398 |
| 6 | 0.00126 | (-0.00010,0.00262) | 0.069 | 0.00088 | (0.00017,0.00158) | 0.015 | -0.00032 | (-0.00131,0.00068) | 0.532 |
| 7 | -0.00113 | (-0.00248,0.00022) | 0.101 | -0.0004 | (-0.00110,0.00030) | 0.265 | -0.00017 | (-0.00115,0.00082) | 0.741 |
| 8 | -0.00177 | (-0.00313,-0.00041) | 0.011 | -0.00043 | (-0.00114,0.00027) | 0.229 | 0.00002 | (-0.00097,0.00102) | 0.962 |
| 9 | -0.00048 | (-0.00184,0.00088) | 0.487 | -0.00067 | (-0.00138,0.00003) | 0.061 | 0.00074 | (-0.00025,0.00173) | 0.142 |
| 10 | -0.00055 | (-0.00191,0.00081) | 0.430 | -0.00042 | (-0.00113,0.00029) | 0.244 | -0.00038 | (-0.00137,0.00061) | 0.451 |
|  |  |  |  |  |  |  |  |  |  |
|  | **VDBP rs4588** | | | **VDBP rs7041** | | | **VDBP rs1155563** | | |
| 1 | -0.0003 | (-0.00173,0.00112) | 0.675 | -0.00054 | (-0.00184,0.00077) | 0.422 | -0.00029 | (-0.00126,0.00069) | 0.564 |
| 2 | -0.00044 | (-0.00184,0.00095) | 0.532 | -0.00036 | (-0.00164,0.00091) | 0.576 | 0.00046 | (-0.00052,0.00144) | 0.353 |
| 3 | 0.00095 | (-0.00047,0.00236) | 0.189 | 0.00001 | (-0.00129,0.00130) | 0.991 | 0.00097 | (-0.00000,0.00195) | 0.051 |
| 4 | 0.00043 | (-0.00096,0.00182) | 0.541 | 0.0001 | (-0.00117,0.00138) | 0.872 | 0.00014 | (-0.00084,0.00113) | 0.772 |
| 5 | 0.0003 | (-0.00112,0.00171) | 0.680 | 0.00068 | (-0.00063,0.00198) | 0.309 | 0.00025 | (-0.00073,0.00123) | 0.617 |
| 6 | -0.00023 | (-0.00167,0.00120) | 0.752 | -0.00127 | (-0.00259,0.00005) | 0.059 | -0.00027 | (-0.00126,0.00071) | 0.587 |
| 7 | 0.00017 | (-0.00124,0.00158) | 0.812 | 0.00041 | (-0.00088,0.00170) | 0.531 | -0.00023 | (-0.00121,0.00074) | 0.639 |
| 8 | 0.00081 | (-0.00058,0.00220) | 0.253 | 0.00005 | (-0.00123,0.00132) | 0.943 | 0.00003 | (-0.00096,0.00101) | 0.956 |
| 9 | 0.00102 | (-0.00039,0.00242) | 0.156 | 0.00036 | (-0.00094,0.00165) | 0.589 | 0.0008 | (-0.00018,0.00178) | 0.112 |
| 10 | -0.00032 | (-0.00173,0.00109) | 0.658 | 0.00079 | (-0.00050,0.00208) | 0.231 | -0.00092 | (-0.00190,0.00006) | 0.067 |
|  |  |  |  |  |  |  |  |  |  |
|  | **CYP24A1 rs6013897** | | | **CYP27B1 rs703842** | | | **CYP27B1 rs10877012** | | |
| 1 | -0.00038 | (-0.00154,0.00078) | 0.519 | 0.00107 | (0.00010,0.00204) | 0.031 | 0.00072 | (-0.00070,0.00213) | 0.319 |
| 2 | 0.00049 | (-0.00068,0.00165) | 0.410 | -0.00025 | (-0.00122,0.00073) | 0.618 | 0.00054 | (-0.00083,0.00192) | 0.439 |
| 3 | 0.0002 | (-0.00096,0.00136) | 0.733 | -0.00027 | (-0.00124,0.00070) | 0.581 | -0.00077 | (-0.00216,0.00063) | 0.281 |
| 4 | -0.00003 | (-0.00120,0.00113) | 0.954 | 0.00001 | (-0.00097,0.00098) | 0.990 | 0.00008 | (-0.00129,0.00145) | 0.908 |
| 5 | -0.00049 | (-0.00165,0.00068) | 0.412 | 0.00056 | (-0.00042,0.00153) | 0.264 | 0.00047 | (-0.00092,0.00186) | 0.505 |
| 6 | 0.00053 | (-0.00063,0.00170) | 0.370 | -0.00115 | (-0.00212,-0.00017) | 0.022 | -0.00156 | (-0.00297,-0.00015) | 0.031 |
| 7 | -0.00056 | (-0.00172,0.00060) | 0.343 | 0.00153 | (0.00056,0.00250) | 0.002 | 0.00111 | (-0.00028,0.00251) | 0.116 |
| 8 | -0.00004 | (-0.00120,0.00113) | 0.951 | -0.00125 | (-0.00222,-0.00027) | 0.012 | -0.00155 | (-0.00291,-0.00018) | 0.027 |
| 9 | -0.00019 | (-0.00136,0.00097) | 0.747 | 0.00056 | (-0.00042,0.00153) | 0.264 | 0.00051 | (-0.00088,0.00190) | 0.469 |
| 10 | -0.00081 | (-0.00197,0.00036) | 0.176 | -0.00005 | (-0.00103,0.00093) | 0.918 | 0.00034 | (-0.00105,0.00173) | 0.629 |
|  |  |  |  |  |  |  |  |  |  |
|  | **Metabolism score^d^** | | | **VDR ApaI** | | | **VDR BsmI** | | |
| 1 | 0.00059 | (-0.00051,0.00168) | 0.294 | -0.0018 | (-0.00308,-0.00052) | 0.006 | 0.00113 | (-0.00020,0.00245) | 0.095 |
| 2 | 0.00035 | (-0.00072,0.00142) | 0.519 | 0.00063 | (-0.00063,0.00189) | 0.326 | -0.00026 | (-0.00155,0.00104) | 0.699 |
| 3 | -0.00011 | (-0.00119,0.00097) | 0.843 | 0.00066 | (-0.00062,0.00194) | 0.310 | -0.00081 | (-0.00213,0.00050) | 0.227 |
| 4 | -0.00017 | (-0.00123,0.00090) | 0.760 | -0.00103 | (-0.00229,0.00022) | 0.105 | 0.00129 | (0.00000,0.00258) | 0.050 |
| 5 | 0.00021 | (-0.00087,0.00129) | 0.699 | -0.00133 | (-0.00262,-0.00005) | 0.042 | 0.00084 | (-0.00048,0.00216) | 0.214 |
| 6 | -0.00088 | (-0.00198,0.00022) | 0.116 | 0.00002 | (-0.00128,0.00132) | 0.972 | -0.00027 | (-0.00161,0.00106) | 0.689 |
| 7 | 0.00002 | (-0.00106,0.00110) | 0.969 | -0.00007 | (-0.00134,0.00120) | 0.916 | 0.00028 | (-0.00103,0.00159) | 0.671 |
| 8 | -0.00069 | (-0.00175,0.00037) | 0.203 | 0.00061 | (-0.00065,0.00187) | 0.341 | 0.00011 | (-0.00118,0.00140) | 0.870 |
| 9 | 0.00015 | (-0.00093,0.00123) | 0.787 | 0.00088 | (-0.00040,0.00215) | 0.177 | -0.00089 | (-0.00220,0.00042) | 0.184 |
| 10 | -0.00035 | (-0.00143,0.00073) | 0.526 | 0.00035 | (-0.00092,0.00163) | 0.587 | -0.00023 | (-0.00154,0.00108) | 0.732 |
|  |  |  |  |  |  |  |  |  |  |
|  | **VDR FokI** | | | **VDR TaqI** | | | **VDR Cdx2** | | |
| 1 | 0.0004 | (-0.00100,0.00180) | 0.577 | 0.00114 | (-0.00019,0.00248) | 0.093 | 0.0021 | (0.00053,0.00367) | 0.009 |
| 2 | 0.00021 | (-0.00116,0.00158) | 0.766 | -0.00027 | (-0.00157,0.00104) | 0.686 | 0.00008 | (-0.00146,0.00162) | 0.916 |
| 3 | 0.00043 | (-0.00095,0.00182) | 0.541 | -0.00078 | (-0.00210,0.00054) | 0.247 | -0.0006 | (-0.00216,0.00096) | 0.449 |
| 4 | -0.00075 | (-0.00210,0.00061) | 0.279 | 0.00126 | (-0.00003,0.00256) | 0.056 | 0.00011 | (-0.00141,0.00164) | 0.884 |
| 5 | -0.00031 | (-0.00170,0.00108) | 0.662 | 0.0008 | (-0.00052,0.00213) | 0.235 | -0.00035 | (-0.00191,0.00121) | 0.661 |
| 6 | 0.00008 | (-0.00133,0.00148) | 0.913 | 0.00004 | (-0.00130,0.00139) | 0.952 | -0.00026 | (-0.00184,0.00132) | 0.744 |
| 7 | -0.00038 | (-0.00176,0.00100) | 0.586 | 0.00047 | (-0.00084,0.00179) | 0.482 | 0.00039 | (-0.00116,0.00194) | 0.622 |
| 8 | -0.00045 | (-0.00182,0.00091) | 0.514 | -0.00024 | (-0.00154,0.00106) | 0.715 | -0.00069 | (-0.00222,0.00084) | 0.378 |
| 9 | 0.00023 | (-0.00115,0.00161) | 0.746 | -0.00119 | (-0.00251,0.00012) | 0.075 | 0.00034 | (-0.00121,0.00189) | 0.666 |
| 10 | 0.00122 | (-0.00016,0.00260) | 0.084 | -0.00047 | (-0.00179,0.00085) | 0.487 | -0.00072 | (-0.00227,0.00084) | 0.365 |
| ^a^ Principal components calculated with the program Eigenstrat | | | | | | | | |  |
| ^b^ Calculated using regression, unadjusted. | | | | | | | | |  |
| ^c^ Synthesis score: rs10741657, rs12785878 | | | | | | | | |  |
| ^d^ Metabolism score: rs6013897, rs10877012. | | | | | | | | |  |
| CI=confidence interval. | | | | | | | | |  |
